# Developmental Exposure to Endocrine Disrupting Chemicals and Type 1 Diabetes Mellitus

**DOI:** 10.3389/fendo.2018.00513

**Published:** 2018-09-03

**Authors:** Sarah G. Howard

**Affiliations:** Diabetes and Environment Program, Commonweal, Bolinas, CA, United States

**Keywords:** type 1 diabetes, endocrine disruptors, arsenic, BPA, phthalates, persistent organic pollutants, air pollution, DOHaD

## Abstract

Exposure to endocrine disrupting chemicals (EDCs) may have implications for the development of type 1 diabetes mellitus (T1DM), especially if exposure occurs during development. Exposure to EDCs during fetal or early life can disrupt the development of both the immune system and the pancreatic beta cells, potentially increasing susceptibility to T1DM later in life. Developmental exposure to some EDCs can cause immune system dysfunction, increasing the risk of autoimmunity. In addition, developmental exposure to some EDCs can affect beta cell development and function, influencing insulin secretion. These changes may increase stress on the beta cells, and identify them as a target to the immune system. Developmental exposure to EDCs that disrupt metabolism by increasing insulin resistance or obesity may also stress the beta cells. Exposure to these EDCs during development may play a role in the pathogenesis of T1DM, and requires further research.

## Introduction

Type 1 diabetes mellitus (T1DM) is an autoimmune disease characterized by inadequate insulin secretion, in which the insulin-producing pancreatic beta cells are targeted and destroyed by the immune system. In children, the incidence of T1DM began increasing in the mid-Twentieth century simultaneously in numerous industrialized countries, and continued to increase over the following decades (1). This increase has been especially rapid in children under 5 years of age, with an additional trend of diagnosis at earlier ages in life (1, 2). The increase appears to have affected those at moderate genetic risk, as the proportion of children with T1DM who are at high genetic risk has not changed in recent decades (2). The cause of the increase remains unexplained (2).

The development of autoimmunity precedes T1DM, and markers of autoimmunity can appear very early in life, suggesting that environmental exposures in early life play a role in triggering T1DM (2). The autoantibodies associated with T1DM may appear before 6 months of age, and the majority of children who develop T1DM before puberty test positive for autoantibodies before age four (3). Other immunological markers that are associated with the later development of T1DM in children may be present at birth or during mid-gestation, illustrating that even prenatal exposures may play a role in the development of T1DM (4, 5).

The idea that exposure to environmental factors during susceptible developmental periods can affect health later in life is known as the Developmental Origins of Health and Disease (DOHaD) hypothesis. This hypothesis applies to a variety of environmental factors, including exposure to endocrine disrupting chemicals (EDCs). Exposure to EDCs is widespread, and in some cases ubiquitous. Pregnant women are exposed to numerous EDCs, which can cross the placenta, and enter the fetus (6). The developing fetus is particularly vulnerable to these exposures, because bodily systems such as the immune system are developing rapidly in the periods before and after birth (7). Exposures that occur during development may affect the growing fetus and infant in ways that would not occur in adults, and these effects are more persistent (7).

The changes that result from EDC exposures during development may have lifelong ramifications. These changes may be relevant for the development of T1DM later in life. It is hormones that guide development of endocrine glands such as the pancreas (8). Exposure to EDCs during development are linked not only to later-life changes to the immune system, including autoimmunity (7), but also to effects on the developing pancreas, as well as changes in metabolism, including type 2 diabetes mellitus (T2DM), insulin resistance, and obesity (8).

Exposure to some EDCs has been associated with the development of T1DM (9, 10). Yet most studies on T1DM and EDCs do not address exposures during prenatal or early life. Because these developmental exposures can affect the immune system, the pancreatic beta cells, and metabolism, exposure to EDCs during development may play a role in the development of T1DM.

## Developmental exposure to EDCs and T1DM

Very few epidemiological studies have directly examined prenatal exposure to EDCs and later-life T1DM. For example, a study from Sweden found that maternal exposure to higher levels of air pollution during pregnancy was associated with a higher risk of T1DM in the offspring (11). Additional studies on air pollution—which can contain EDCs—have also found links between early childhood exposures and later T1DM (12, 13). However, another study, also from Sweden, found no association between *in utero* levels of two persistent organic pollutants (POPs) and T1DM in childhood (14). In fact, the trend was in the opposite direction, showing a possible protective effect. This finding might be explained by the higher fish intake in Swedes with higher levels of POPs, since the omega three fatty acids found in fish may protect against T1DM. A number of studies have linked T1DM development to exposure to nitrates and related compounds, some with developmental, or childhood exposures (9). Thus, while contradictory, there is some preliminary epidemiological evidence that developmental exposure to some EDCs may affect the later-life risk of T1DM, although more research is clearly needed.

Similar to the epidemiological evidence, there are also only a handful of experimental studies directly evaluating developmental exposure to EDCs and later T1DM. Some of these studies have used non-obese diabetic (NOD) mice as an animal model of T1DM. For example, maternal exposure to bisphenol A (BPA), used in a wide variety of consumer products, accelerated insulitis, and diabetes development in NOD mice offspring, although only at high exposure levels (15). At lower, environmentally relevant exposure levels, when the exposure occurred from conception throughout life, BPA also accelerated diabetes development in NOD mice (16). A mixture of phthalate plasticizers with BPA, however, seemed to counteract the acceleration of diabetes caused by BPA in these mice, although did not dampen the development of insulitis (16). Developmental exposure to perfluoroundecanoic acid (PFUnDA), a replacement for other perfluorinated chemicals (PFCs), also accelerated the development of insulitis in NOD mice (17). Interestingly, additional studies found that environmental chemicals did not accelerate insulitis or diabetes in NOD mice, and while I questioned whether these mice were appropriate for use in testing chemicals in relation to T1DM (10), the differing results may instead be due to the timing of exposure, with developmental exposures showing different effects than adult exposures. Using anotheranimal model, juvenile alligators exposed to tank water with high levels of nitrate (a possible EDC) after hatching developed biomarkers consistent with T1DM, beginning early in life and becoming stronger later in life (18). Thus some laboratory studies show that developmental exposure to EDCs may influence the development of T1DM in animal models, but very few chemicals have been evaluated.

Due to the lack of direct research on developmental exposure to EDCs and T1DM, additional research on endpoints related to T1DM are illuminating, and suggest that indeed these exposures could be important for T1DM. The endpoints of autoimmunity, pancreatic beta cell development, and metabolism may shed additional light on how developmental EDC exposures could contribute to T1DM.

## Developmental exposure to EDCs and the immune system

Exposure to EDCs during development are associated with immune system changes in humans. For example, a number of epidemiological studies found that *in utero* exposure to the EDC arsenic, a common drinking water contaminant, is associated with immunological changes in newborns. Some of these changes are in turn linked to markers that may be relevant for T1DM. For example, prenatal exposure to arsenic is associated with populations of cord blood immune cells linked to the development of autoimmunity, and with epigenetic changes in newborns, some involved in pathways relevant for T1DM and T2DM (19–22). We do not yet know if these immunological changes found at birth are persistent or will have later-life health effects, but numerous epidemiological studies have linked chronic arsenic exposure to the development of T2DM, and arsenic metabolism is associated with T1DM (23).

Experimental studies show that developmental exposure to additional EDCs is linked to the development of autoimmunity in particular. For example, prenatal exposure to 2,3,7,8-tetrachlorodibenzo-*p*-dioxin (TCDD), a POP, promoted later-life autoimmunity in mice (24). Developmental exposure to BPA is linked to various immune system diseases, including autoimmune diseases (25). In fact, BPA can impact essentially all the major cells of the immune system, and can cause a number of effects that are linked to autoimmunity triggers (26). The adult offspring of pregnant rats exposed to low levels of the plasticizer di(2-ethylhexyl) phthalate (DEHP) had epigenetic changes to genes that control the immune response. Interestingly, these changes did not occur when adults were exposed (27). Taken together, these studies illustrate that developmental exposure to various EDCs can affect the development of the immune system in ways that may promote autoimmunity. Some also illustrate that the effects of developmental exposures may be different than those of adult exposures.

## Developmental exposure to EDCs and the pancreas

Autoimmunity is not the only contributor to the development of T1DM. Some environmental factors are suspected to contribute to T1DM by overloading the pancreatic beta cells. According to the “overload hypothesis,” any environmental factor that stresses or overloads the beta cells may sensitize these cells to damage from the immune system and accelerate their demise (28). Interestingly, beta cell stress could perhaps even act a trigger that initiates beta cell autoimmunity. In fact, recent research has found a potential biological mechanism linking beta cell stress to autoimmunity—the discovery of peptides produced by stressed beta cells that can initiate beta cell specific autoimmunity (29). Whether or not beta cell stress plays a role before or after the development of autoimmunity, environmental factors that stress beta cells may play a role in the development of T1DM.

A number of environmental factors can cause beta cell stress, including excess weight, rapid growth, infection, physical or psychological stress or trauma, inflammation, puberty, pregnancy, and insulin resistance. Many of these factors are linked to T2DM or gestational diabetes, and many are also linked to T1DM. An additional, often unrecognized cause of beta cell stress is developmental exposure to EDCs.

Developmental exposure to EDCs can affect the development of the pancreas, the pancreatic islets, and beta cells, leading to changes in insulin secretion. Longitudinal epidemiological studies have found that perinatal EDC exposures are associated with changes in insulin levels in infants, children, or adolescents (30–33). Exposure to EDCs during childhood is also associated with changes to insulin levels or to beta cell function in childhood (33–35). While the specific findings of these studies vary by chemical, sex, timing of exposure, and even pubertal status, they illustrate that both prenatal and early childhood chemical exposures are associated with changes in insulin secretion from beta cells later in life.

While some of these epidemiological studies found that EDC exposure was associated with decreased insulin secretion (30, 31, 33–35), others found exposure associated with increased insulin secretion (30, 32, 33). Both increased and decreased insulin secretion may be important in the development of diabetes. Decreased insulin secretion is characteristic of T1DM, and indicates toxicity to beta cells. Increased insulin secretion is characteristic of early T2DM, and in itself can contribute to the development of insulin resistance and glucose intolerance (36). Both increased and decreased insulin secretion can therefore cause stress on the beta cells.

Experimental studies in rodents show that developmental exposure to numerous chemicals can affect the development of the pancreas and beta cells. For example, exposure to low levels of arsenic throughout life damaged pancreatic beta cells and caused impaired glucose metabolism in adult rats (37). Developmental exposure to air pollutants caused beta cell dysfunction, reduced islet and beta cell size, lowered insulin secretion, and impaired glucose tolerance in adult male mice (38). Developmental exposure to DEHP impaired the pancreas in early life and led to glucose intolerance in adult rat offspring, at exposure levels relevant to high-level human exposures. Laboratory studies therefore show that developmental exposure to EDCs can affect the pancreas in adulthood.

The effects of developmental exposure to EDCs on the pancreas can begin earlier than adulthood, however. In mice, *in utero* exposure to BPA altered islet cell development in the fetal pancreas (39). After birth, *in utero* exposure to environmentally relevant doses of BPA at first led to increased beta cell mass and insulin levels, but later in life led to the same or lower beta cell mass (36). At the time of weaning, developmental exposure to environmentally relevant levels of DEHP led to reduced beta cell mass, lower pancreatic insulin content, and alterations in the expression of genes involved in pancreas development and beta cell function in offspring. In adulthood, exposed female offspring had higher blood glucose levels, impaired glucose tolerance, lower insulin secretion, and lower insulin levels than controls, while males had higher insulin levels (40). Prenatal exposure to DEHP resulted in higher blood glucose levels, impaired insulin and glucose tolerance, impaired insulin secretion and decreased pancreatic insulin content in young rats. Epigenetic mechanisms appeared to play a role, as DEHP exposed offspring also had down-regulated expression of genes involved in the development and function of beta cells (41). These studies illustrate that developmental exposure to various EDCs can affect pancreatic development beginning in the womb and into adulthood.

Interestingly, maternal folate supplementation during pregnancy counteracted the pancreatic effects of BPA (which included disrupted insulin secretion, impaired beta cell morphology, and glucose intolerance) in adult rat offspring (42). In fact, the association between arsenic metabolism and T1DM in humans also depended on folate levels (23), thus illustrating that nutritional status may interact with EDCs to influence their effects.

In addition to these rodent studies, experimental studies on zebrafish embryos, another animal model used to evaluate the effects of EDCs, also show that developmental exposure to numerous chemicals affect the development of the pancreas and beta cells *in utero*. Developmental exposure to a number of chemicals, including arsenic, phthalates, the POPs polychlorinated biphenyls (PCBs), and perfluorooctanesulfonic acid (PFOS) (a PFC), affected the pancreatic development of zebrafish embryos, resulting in flawed development of the islets and beta cells (43–46). For example, exposure to a dioxin-like PCB resulted in inappropriate development of the pancreatic islets and beta cells in zebrafish embryos (46). Exposure to PFOS led to decreased pancreatic islet size and changes in islet morphology in embryos (45). Mono(2-ethylhexyl) phthalate (MEHP), a metabolite of DEHP, affected pancreatic development, reducing beta cell area, and affected gene expression in embryos as well (43). In sum, these experimental studies illustrate that developmental exposure to numerous EDCs can affect the development of the pancreas and specifically the beta cells *in utero*.

Alarmingly, some of the pancreatic effects of chemical exposure during development can be passed down to subsequent generations. In rats, prenatal exposure to DDE (at levels similar to those found in highly exposed humans) led to pancreatic effects not only in the offspring of exposed pregnant mothers, but also in two subsequent generations. These effects included impaired glucose tolerance, abnormal insulin secretion, beta cell dysfunction, and reduced beta cell area, and were transferred through the male germ line (47). Male mice offspring perinatally exposed to environmentally relevant levels of BPA had lower insulin secretion as adults, as well as islet inflammation that persisted into the next generation. The effects of the BPA exposure varied by sex, as well as by exposure level. Mice exposed to the lower exposure level had reduced beta cell mass and increased beta cell death, while those exposed to higher levels had impaired mitochondrial function in beta cells (48). Another study also found that perinatal exposure to low levels of BPA caused beta cell dysfunction and lower insulin secretion in two generations of adult male rat offspring (49). These studies raise the possibility that developmental exposures to EDCs can have effects on the pancreas that persist not only into adulthood, but into subsequent generations. Whether developmental EDC exposures in prior generations can affect the risk of diabetes in humans is unknown, although there is human evidence that developmental exposure to famine can increase the risk of hyperglycemia in subsequent generations (50).

Experimental studies thus show that developmental exposure to EDCs can affect the development of the pancreas and beta cells, influencing insulin secretion, beginning in the womb and continuing into adulthood. These laboratory results are supported by epidemiological studies showing associations between developmental exposure to EDCs and insulin secretion later in life. As beta cell stress is a potential contributor to T1DM, exposure to EDCs in prenatal and early life should be thoroughly analyzed in relation to the development of T1DM.

## Developmental exposure to EDCs and additional effects

In addition to autoimmunity and pancreatic development, developmental exposure to EDCs can have additional effects that may be related to T1DM. For example, EDCs can affect the permeability, inflammation, and microbiota of the intestine (51), which in turn are linked to T1DM (2). EDCs are also linked to an increased risk of vitamin D deficiency (52), a risk factor also associated with T1DM development (2).

Exposure to EDCs is strongly linked to the development of T2DM, insulin resistance, and obesity, in both experimental and epidemiological studies (8). In humans, developmental exposures to numerous EDCs are linked to metabolic changes in infants and children, including insulin resistance and obesity-related outcomes (53). While insulin resistance and obesity are clearly important in the development of T2DM, there is some evidence that these metabolic factors may also play a role in the development of T1DM. For example, insulin resistance, and obesity may contribute to the development of T1DM by stressing the pancreatic beta cells and accelerating their loss (2).

## Conclusion

As early signs of T1DM may be apparent in early childhood, or even before birth, prenatal and early life exposures likely play a role in the development of T1DM, supporting the DOHaD hypothesis. Pregnant women are exposed to multiple EDCs, and these chemicals can cross the placenta and enter the fetus, potentially affecting the later risk of disease in the offspring (6). Developmental exposure to EDCs is under-researched in T1DM. Only a small number of studies have addressed the potential role of developmental exposure to EDCs in T1DM, and only a small number of chemicals have been analyzed—almost none in long-term prospective studies (9, 10). These exposures, however, should be a prime candidate for consideration in the pathogenesis of T1DM. Developmental exposure to EDCs can have effects that could be important in the development of T1DM, including promoting autoimmunity, affecting the development of the pancreas and beta cells specifically, influencing insulin secretion, and disrupting metabolism. Experimental studies show that these effects can appear as early as in the womb, last into adulthood, and sometimes even be passed on to subsequent generations. Certain chemicals, including for example arsenic, BPA, and DEHP, are linked to changes in the development of both the immune system and the pancreas, and should be of prime interest in additional research. The role of developmental exposure to EDCs as contributors to the development of T1DM should be an urgent focus of research, and may help provide an eventual pathway for prevention.

## Author contributions

The author confirms being the sole contributor of this work and approved it for publication.

### Conflict of interest statement

The author declares that the research was conducted in the absence of any commercial or financial relationships that could be construed as a potential conflict of interest.

## Abbreviations

BPA: bisphenol A
DOHaD: Developmental Origins of Health and Disease
DEHP: di(2-ethylhexyl) phthalate
EDC: endocrine disrupting chemical
MEHP: mono(2-ethylhexyl) phthalate
NOD: non-obese diabetic
PCB: polychlorinated biphenyl
POP: persistent organic pollutant
PFC: perfluorinated chemical
PFOS: perfluorooctanesulfonic acid
PFUnDA: perfluoroundecanoic acid
T1DM: type 1 diabetes mellitus
T2DM: type 2 diabetes mellitus
TCDD, 2, 3, 7: 8-tetrachlorodibenzo-*p*-dioxin.
